# Circulating endothelial progenitor cells and inflammatory markers in type 1 diabetes after an acute session of aerobic exercise

**DOI:** 10.20945/2359-4292-2023-0499

**Published:** 2024-11-06

**Authors:** Patrícia Martins Bock, Raíssa Borges Monteiro, Clara Krummenauer Maraschin, Ana Paula Alegretti, Mariela Granero Farias, Fabiane Spagnol, Patricia Luciana da Costa Lopez, Lucas Porto Santos, Lucas Helal, Ruy Silveira Moraes, Daniel Umpierre, Beatriz D. Schaan

**Affiliations:** 1 Instituto Nacional de Ciência e Tecnologia para Avaliação de Tecnologias em Saúde Hospital de Clínicas de Porto Alegre Porto Alegre RS Brasil Instituto Nacional de Ciência e Tecnologia para Avaliação de Tecnologias em Saúde (IATS) – CNPq/Brasil, Hospital de Clínicas de Porto Alegre, Porto Alegre, RS, Brasil; 2 Universidade Federal do Rio Grande Rio Grande RS Brasil Universidade Federal do Rio Grande, Rio Grande, RS, Brasil; 3 Centro de Pesquisa Clínica/Centro de Pesquisa Experimental Hospital de Clínicas de Porto Alegre Porto Alegre RS Brasil Centro de Pesquisa Clínica/Centro de Pesquisa Experimental, Hospital de Clínicas de Porto Alegre, Porto Alegre, RS, Brasil; 4 Universidade Federal do Rio Grande do Sul Faculdade de Medicina Departamento de Clínica Médica Porto Alegre RS Brasil Universidade Federal do Rio Grande do Sul, Faculdade de Medicina, Departamento de Clínica Médica, Programa de Pós-graduação em Ciências Médicas: Endocrinologia, Porto Alegre, RS, Brasil; 5 Serviço de Diagnóstico Laboratorial Hospital de Clínicas de Porto Alegre Porto Alegre RS Brasil Serviço de Diagnóstico Laboratorial (SDLab), Hospital de Clínicas de Porto Alegre, Porto Alegre, RS, Brasil; 6 Universidade Federal do Rio Grande do Sul Porto Alegre RS Brasil Universidade Federal do Rio Grande do Sul, Programa de Pós-graduação em Ciências da Saúde: Cardiologia, Porto Alegre, RS, Brasil

**Keywords:** Tumor necrosis factor-alpha, interleukin 6, chemokine receptors, endothelium

## Abstract

**Objective:**

To determine circulating endothelial progenitor cells (EPC) counts and levels of inflammatory markers in individuals with and without type 1 diabetes mellitus (T1DM) in response to an intense aerobic exercise session.

**Subjects and methods:**

In total, 15 adult men with T1DM and 15 healthy individuals underwent a 30-minute aerobic exercise session on a cycle ergometer at 60% of the peak heart rate. The EPC count (CD45^dim^/CD34^+^/KDR^+^), tumor necrosis factor-alpha (TNF-α) and interleukin 6 (IL-6) levels were measured before and 60 minutes after the session.

**Results:**

We found no difference within or between groups regarding EPC counts before and after the aerobic exercise: healthy individuals, 0.02% change (95% confidence interval [CI] −0.04%-0.08%); individuals with T1DM, 0.00% (95%CI −0.01%-0.01%). We also found no difference in TNF-α levels before and after exercise in healthy individuals (210.2, interquartile range [IQR] 142.1-401.2 pg/mL and 191.3, IQR 136.4-350.5 pg/mL, respectively) and in patients with T1DM (463.8, IQR 201.4-4306.0 pg/mL and 482.7, IQR 143.8-4304.3 pg/mL, respectively). Similarly, no difference in IL-6 levels was observed before and after exercise in healthy individuals (148.2, IQR 147.5-148.6 pg/mL and 148.2, IQR 147.7-148.6 pg/mL, respectively) and individuals with T1DM (147.2, IQR 145.9-147.7 pg/mL and 147.2, IQR 146.8-147.8 pg/mL, respectively).

**Conclusions:**

Patients with T1DM and healthy controls had comparable EPC responses to aerobic exercise, most likely due to the absence of a chronic inflammatory state.

## INTRODUCTION

The endothelium is a key regulator of vascular homeostasis (1). The integrity of the endothelium depends on its ability to repair and renew through the replication of adjacent endothelial cells and circulating endothelial progenitor cells (EPCs) (2). These cells are recruited from the bone marrow and released into the circulation when endothelial repair is required. This process is regulated by angiogenic growth factors (*e.g.*, vascular endothelial growth factor [VEGF] and angiopoietin) and chemokines (*e.g.*, stromal cell-derived factor-1α [SDF-1α]). Notably, SDF-1α can exert its role through binding to C-X-C chemokine receptor 4 (CXCR-4) (3), which leads EPCs to differentiate into endothelial cells (4). Endothelial dysfunction is an early predictor of atherosclerosis, and one of the most important stimuli for this impaired vascular function is a proinflammatory environment (5). Tumor necrosis factor-alpha (TNF-α) is an important proinflammatory cytokine that may induce EPC proliferation and reduce migratory capacity (6).

Low circulating levels of EPCs can be a marker of endothelial dysfunction in diabetes (7,8). Cardiovascular risk factors (including diabetes) are associated with reduced mobilization and differentiation of EPCs (9). Impaired mobilization or depletion of these cells contributes to compromised endothelial cell repair (10). Additionally, reduced levels of circulating stem cells predict the occurrence of cardiovascular events in patients with type 2 diabetes (11), while diabetes is associated with impaired stem and progenitor cell mobilization after direct bone marrow stimulation (12).

Levels of circulating EPCs can be increased in patients with optimal glycemic control (13), suggesting that exercise – an effective strategy for glycemic control (14) – may improve endothelial function. Indeed, exercise can increase circulating EPCs both acutely and chronically and could be a potential strategy for improving their mobilization and function (15,16). We have previously shown that a 40-minute aerobic exercise bout (60% of peak oxygen uptake [VO_2_]) acutely leads to lower levels of circulating EPCs in healthy individuals (17). Given the need for arterial repair after aerobic exercise, the lower EPC levels observed may be due to EPC distribution to sites of vascular injury, an effect that we did not observe in patients with type 1 diabetes mellitus (T1DM) in our previous study (17). This observation supports the hypothesis of a blunted endothelial regenerating capacity in T1DM (17). The same has been shown in individuals with type 2 diabetes, who have lower EPC count than those with normal glucose tolerance. After a 30-minute acute bout of exercise at submaximal intensity, circulating EPC counts increased in the group with normal glucose tolerance but not in the group with type 2 diabetes (18). While it is unclear how methodological differences (such as exercise type and duration) elicit different EPC responses in healthy individuals, both studies demonstrated impaired EPC responses in diabetes.

To further understand the EPC mobilization in individuals with diabetes under exercise conditions, we conducted this nonrandomized, controlled trial measuring circulating EPC count using flow cytometry in patients with T1DM and healthy individuals and estimating EPC populations and inflammatory markers in response to an acute session of aerobic exercise.

## SUBJECTS AND METHODS

In this nonrandomized, controlled trial, individuals with T1DM were recruited from the endocrinology outpatient clinic of a tertiary teaching hospital and through a website. The inclusion criteria were male individuals aged 18 to 65 years with a diagnosis of T1DM. The exclusion criteria were chronic diabetic complications that could hinder the participant's ability to perform the exercise protocol, including severe autonomic neuropathy, overt diabetic nephropathy, chronic renal failure (glomerular filtration rate below 30 mL/hour), limb amputation, disabling peripheral artery disease, coronary artery disease, and heart failure. For the control group, we recruited healthy sex and age-matched individuals who were not obese or smokers.

The sample size, which was calculated *a priori*, was intended to ensure that the analysis of circulating EPCs would detect a difference of at least 3.9 cells/10^6^ (standard deviation [SD] 3.0 cells/10^6^) in white blood cells (WBC) between the groups after the intervention. This resulted in 13 participants to achieve a 90% power and a 5% type I error rate (19). To account for an estimated 10% rate of participant loss or refusal, we planned to enroll 15 participants in each group, for a total of 30 study participants.

The protocol of the study was approved by the Institutional Review Board of the *Hospital de Clínicas de Porto Alegre*, Brazil (tracking number: 52451616.5.0000.5327). The study was conducted in accordance with the Declaration of Helsinki. The present reporting of the study was structured according to the Transparent Reporting of Evaluations with Nonrandomized Designs (TREND) guideline for nonrandomized clinical trials (20).

The participants underwent clinical and laboratory evaluations that included the assessment of clinical profile, measurement of blood pressure levels, and collection of fasting blood samples for measurement of levels of plasma glucose, lipids (cholesterol and HDL cholesterol), and glycated hemoglobin (HbA1c). Participants’ weights were measured using a calibrated digital scale (Welmy, São Paulo, Brazil) with a maximum capacity of 150 kg and precision of 100 g. Their heights were measured with a vertical stadiometer attached to the scale, which had a maximum measuring height of 2 meters and a precision of 0.1 cm. The participants’ levels of physical activity were assessed using the Brazilian validated version of the International Physical Activity Questionnaire – long form (IPAQ).

After this evaluation, the participants underwent a self-limited maximal exercise test on a cycle ergometer to determine their peak VO_2_ and peak heart rate, supervised by a team of experienced exercise physiologists and cardiologists. The test was conducted in increments of 15-20 watts/minute, with a cadence of 60-70 rpm, until the participant reached exhaustion. The VO_2_ and carbon dioxide production (VCO_2_) were measured using a breath-by-breath computerized gas exchange system (Oxycon Delta; VIASYS Healthcare GmbH, Jaeger, Germany) and analyzed using a 20-second averaging signal. Peak VO_2_ and peak heart rate were defined as the highest values reached in the last 30 seconds of exercise testing.

Two weeks after undergoing clinical and laboratory evaluations, the participants arrived at the laboratory at approximately 8:00 am to perform an aerobic exercise session on a cycle ergometer. After a 5-minute warm-up, they exercised for 30 minutes at 60% of their peak heart rate, as determined in the maximal exercise test, followed by a 5-minute cool-down period. The intensity of the exercise session was monitored by the participants’ heart rate (Polar F1 TM; Polar Electro Oy, Helsinki, Finland) and perceived exertion. Blood samples were collected 5 minutes before and 60 minutes after the exercise session for measurement of levels of TNF-α, IL-6, and circulating progenitor cells (CPCs). The participants were instructed to abstain from consuming caffeine and engaging in physical exercise on the day prior to the self-limited maximal exercise test, exercise session, and blood sample collection.

To prevent hypoglycemia, the participants with T1DM were instructed to refrain from using their short-acting insulin dose and to maintain the same long-acting insulin dose on the mornings of the cardiopulmonary exercise tests and exercise sessions. Before the exercise sessions, capillary blood glucose was checked in all participants with the use of a self-monitoring of blood glucose (SMBG) device (Accu-Chek, Roche Diabetes Care, Basel, Switzerland), and a snack was provided to participants with T1DM who had blood glucose levels ≤150 mg/dL (of note, only one individual with T1DM received a snack before the exercise session) followed by glucose levels recheck 15 minutes later. The exercise sessions started only when glucose levels were between 150 mg/dL and 250 mg/dL. In the case of glucose levels ≥ 250 mg/dL, the participants’ urine was tested for the presence of ketones (ComboStik, Gyung-Nam, Korea), and the exercise was only started when ketones were not present. Capillary blood glucose levels were measured in both the T1DM and control groups following exercise, and all participants were provided with a snack. Venous blood samples were collected from the antecubital vein into ethylenediaminetetraacetic acid (EDTA)-coated tubes and centrifuged for 10 minutes at 1,000 g for plasma separation. The plasma was then stored at −80 °C for further analyses.

Plasma levels of HbA1c were analyzed using ion-exchange high-performance liquid chromatography (HPLC; Variant II Turbo, Bio-Rad; CA, USA). Plasma levels of glucose were analyzed using the glucose oxidase method (UV; Alinity C, Abbott Laboratories, Chicago, IL, USA), and those of total cholesterol and HDL cholesterol were analyzed using the enzymatic-colorimetric method (Alinity C, Abbott Laboratories). Blood count was analyzed using Sysmex XN10 (Sysmex, Kobe, Japan). A colorimetric enzymatic immunoassay was used to determine the levels of TNF-α (Human TNF-α ELISA Kit [RAB0476], Sigma-Aldrich, St. Louis, MI, USA) and IL-6 (Human IL-6 ELISA Kit [RAB0306], Sigma-Aldrich).

Flow cytometry was used to identify CPCs based on the surface expression of the hematopoietic stem cell markers CD34 and CD133. Notably, CPCs have different populations with different phenotypes, including EPCs. Human EPCs express CD34 and exhibit high membrane expression levels of vascular endothelial growth factor receptor 2 (VEGFR-2), also known as kinase insert domain receptor (KDR) or CD309, a surface marker of the endothelial lineage. They show low or absent expression of the pan-leukocyte marker CD45, as endothelial cells are typically negative for the CD45 antigen. We gated the cells based on forward scatter (FSC) and side scatter (SSC) parameters to exclude debris from the analysis. Subsequently, we used FSC-A and FSC-H to select singlets. The CD34^+^/KDR^+^/CD45^dim/−^ immunophenotype was used due to its better sensitivity, specificity, and reliability in quantifying EPCs in the clinical setting, while CXCR-4 (also known as CD184) was used to evaluate EPC mobilization (21). Total endothelial cells (TECs) were identified by the KDR^+^/CD45^dim/−^ immunophenotype (expressed as a percentage of WBCs) and were further subdivided into CD133^+^/CD34^−^, CD133^+^/CD34^+^, CD133^−^/CD34^+^, and CD133^−^/CD34^−^ cells (expressed as a percentage of TECs). Additionally, total circulating CD34^+^ cells were expressed as a percentage of WBCs. The EPCs were identified by the CD34^+^/KDR^+^/CD45^dim/−^ immunophenotype, while the CD34^+^/KDR^+^/CD45^dim/−^/CXCR4^+^ immunophenotype was used to assess EPC mobilization (expressed as a percentage of EPCs) (21).

**Figure 1:**
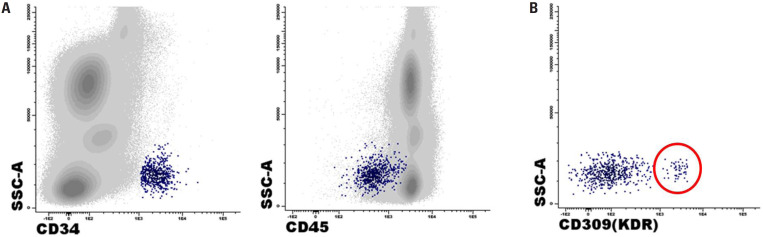
Gating strategy for CD34^+^/CD45^dim/−^ (panel a) and KDR^+^ (panel b) cells.

For immunophenotyping, TECs and EPCs were analyzed using whole blood and the following fluorochrome-conjugated antibody reagents: CD45 FITC (clone 2D1), CD34 PE (clone 8G12), CD309 Alexa Fluor (clone 89106), and CD184 BV421 (clone 12G5) from Becton Dickson (BD Biosciences, San Jose, CA, USA) and CD133 PerCP-eFluor (clone TMP4) from Invitrogen (Life Technologies, CA, USA). The samples were processed according to the EuroFlow erythrocyte bulk-lysis protocol (22) and were subsequently stained using the regular EuroFlow protocol (23). At least 2 million total events were acquired using a FACSCanto II flow cytometer (BD Biosciences) and analyzed using Infinicyt, version 1.7 (Cytognos, Salamanca, Spain) (24). To compensate for the antibodies used in this study, pure cells and cells labeled with each antibody in the panel were acquired individually, using normal cells or beads in the case of rare expression. For each sample analyzed, the negative population of the marker in question was used as a reference to define the gating limits and identify when the target population became positive. A fixed and equal value was not applied to all samples. Populations were not arbitrarily gated; instead, they were analyzed as a whole. A blinded researcher analyzed the samples. Figure 1 shows the gating strategy for CD34^+^/KDR^+^/CD45^dim/-^ cells.

### Statistical analysis

The data were analyzed using the software Statistical Package for the Social Sciences (SPSS) for Windows, version 18.0 (SPSS Inc., Chicago, IL, USA). The data distribution was evaluated for normality using the Shapiro-Wilk test. Values are presented as mean ± SD for variables with a normal distribution; otherwise, median and interquartile range (percentiles 25-75) are provided. Categorical variables are shown as numbers (percentages). Baseline comparisons were tested using Student's *t* test or Pearson's chi-square test (p ≤ 0.05). The effect of the exercise on primary and secondary outcomes was estimated using generalized estimating equations (GEE) followed by Bonferroni's *post hoc* test (p < 0.05).

## RESULTS

**Figure 2:**
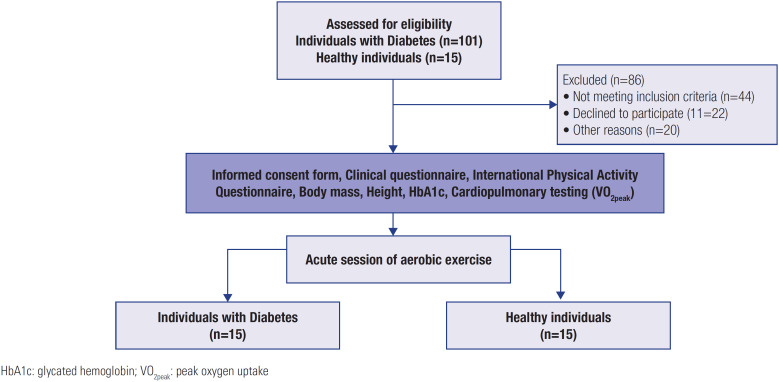
Flowchart of the participants’ selection process.

The study included 15 patients with T1DM and 15 healthy individuals. Figure 2 shows a flowchart of the participants’ selection process at each study stage.

Table 1 shows the clinical and laboratory profiles of the participants. Individuals in both groups had comparable ages, body mass index (BMI) values, total cholesterol and HDL cholesterol levels, blood pressure and heart rate measurements, and physical activity levels. Participants’ ages ranged from 24 to 57 years, and 50% of the individuals in both groups were overweight.

**Table 1:** Clinical and laboratory characteristics of the study participants

Characteristic		Healthy individuals (n = 15)	Individuals with T1DM (n = 15)	P
Age (years)		32 ± 5.4	36 ± 7.6	0.18
Diabetes duration (years)		–	17.0 (10-24)	–
BMI (kg/m²)		25.5 ± 4.5	24.9 ± 4.5	0.73
Insulin				
	NPH	–	6 (40.0)	–
	Glargine	–	5 (33.3)	–
	Regular	–	3 (20.0)	–
	Lispro	–	7 (46.7)	–
	Aspart	–	3 (20.0)	–
Laboratory characteristics				
	Fasting plasma glucose (mg/dL)	88 ± 11.2	197.3 ± 90.0	<0.01
	HbA1c (%)	5.0 ± 0.3	7.9 ± 1.5	<0.01
	Total cholesterol (mg/dL)	175.1 ± 41.6	172.9 ± 30.7	0.87
	HDL cholesterol (mg/dL)	51.8 ± 6.9	50.3 ± 12.0	0.67
Systolic blood pressure (mmHg)		120 (110-122)	131 (112-139)	0.33
Diastolic blood pressure (mmHg)		80 (70-80)	80 (78-87)	0.46
Heart rate (bpm) at rest		76.9 ± 8.7	85.6 ± 13.4	0.05
Heart rate (bpm) peak		179.8 ± 11.2	167.4 ± 21.1	0.06
Peak VO_2_ (mL/kg/min)		37.6 ± 8.4	29.1 ± 10. 9	0.03
IPAQ				
	Insufficiently active	0 (0)	4 (13.3)	0.09
	Sufficiently active	7 (23.3)	6 (20)	
	Very active	8 (26.7)	5 (16.7)	

Abbreviations: BMI, body mass index; HbA1c: glycated hemoglobin; IPAQ, International Physical Activity Questionnaire (long form); NPH, neutral protamine Hagedorn; peak VO_2_, peak oxygen uptake; T1DM, type 1 diabetes mellitus. Continuous variables are expressed as mean ± standard deviation or median (interquartile range [percentiles 25-75]). Categorical variables are expressed as numbers (%). Comparisons were analyzed using Pearson's chi-square test or Student's *t* test.

Fasting plasma glucose and HbA1c levels were higher in individuals with T1DM than in healthy controls, while VO_2_ peak was higher in healthy individuals. All patients with T1DM were using multiple daily insulin injections (NPH or glargine combined with regular insulin or aspart/lispro).

Capillary blood glucose levels measured before and immediately after exercise were higher in patients with T1DM (189 ± 64 mg/dL and 110 ± 58 mg/dL, respectively) than in healthy individuals (96 ± 13 mg/dL and 77 ± 13 mg/dL, respectively; p group < 0.001, p time < 0.001, p interaction = 0.002).

Table 2 shows the blood count values before and after the aerobic exercise session in both groups. After exercise, WBC and neutrophil counts increased in both groups. No differences were observed within and between groups regarding hematocrit and lymphocyte counts before and after exercise, while monocyte counts increased in individuals with T1DM after the exercise.

**Table 2:** Blood count values before and after the aerobic exercise session

		Healthy individuals (n=15)		Individuals with T1DM (n=15)		P Group	P Time	P Interaction
		Before	After	Before	After			
Hematocrit (%)		43.9 ± 2.1	44.4 ± 3.5	43.6 ± 4.1	44.4 ± 44.6	0.881	0.082	0.597
	Change		0.4 (-0.9–1.8)		0.8 (0.1-1.5)			
White blood cells (x 10^3^/µL)		5.7 ± 0.9	6.5 ± 1.5	5.9 ± 1.6	7.0 ± 1.8	0.412	<0.001	0.519
	Change		0.8 (0.1-1.4)		1.0 (0.43-1.6)			
Neutrophils (x 10^3^/µL)		3.1 ± 0.8	4.0 ± 1.4	3.6 ± 1.8	4.5 ± 1.7	0.347	<0.001	0.956
	Change		0.9 (0.3-1.4)		0.9 (0.3-1.4)			
Lymphocytes (x 10^3^/µL)		1.9 ± 0.5	1.8 ± 0.4	1.6 ± 0.3	1.8 ± 0.4	0.353	0.911	0.177
	Change		-0.1 (-0.3–0.1)		0.1 (-0.1–0.1)			
Monocytes (x 10^3^/µL)		0.5 ± 0.1	0.5 ± 0.1	0.48 ± 0.1	0.5 ± 0.1*	0.397	0.400	0.020
	Change		-0.0 (-0.1– 0.0)		0.1 (0.0-0.1)			

The parameters were evaluated before and after the aerobic exercise session. Data are presented as mean ± standard deviation. Comparisons were evaluated using generalized estimating equations (GEE) with Bonferroni correction. Change means difference from post-exercise to pre-exercise (95% confidence interval).

* P < 0.001 *versus pre*-exercise values. Abbreviation: T1DM, type 1 diabetes mellitus.

No differences occurred within or between groups regarding EPCs, TECs, and total CD34^+^ cells before and after the aerobic exercise sessions, as shown in Table 3.

**Table 3:** Endothelial progenitor, total endothelial, and total circulating progenitor cells before and after the aerobic exercise session

		N	Healthy individuals		Individuals with type 1 diabetes mellitus		P Group	P Time	P Interaction
			Before	After	Before	After			
EPC (CD34^+^/KDR^+^/CD45^dim/-^)		24	0.01 ± 0.01	0.03 ± 0.03	0.01 ± 0.00	0.01 ± 0.00	0.151	0.379	0.215
	Change			0.02 (-0.04–0.08)		-0.00 (-0.01–0.01)			
CXCR4^+^		10	0.06 ± 0.04	0.00 ± 0.00	0.00 ± 0.00	0.00 ± 0.00	0.151	0.184	0.191
	Change			-0.05 (-0.13–0.03)		-0.00 (-0.00–0.00)			
TEC (KDR^+^/CD45^dim/-^)		23	0.03 ± 0.02	0.02 ± 0.04	0.03 ± 0.03	0.03 ± 0.03	0.937	0.578	0.602
				-0.01 (-0.01–0.07)		-0.00 (-0.01–0.05)			
CD133+/CD34^-^		21	27.96 ± 8.96	36.08 ± 8.14	38.15 ± 8.10	40.28 ± 9.50	0.426	0.558	0.704
	Change			8.12 (0.98-15.26)		2.57 (-8.91–14.04)			
CD133^+^ /CD34^+^		21	28.44 ± 7.21	25.23 ± 5.13	30.03 ± 7.48	33.74 ± 10.42	0.596	0.940	0.735
	Change			-3.21 (-13.38–6.96)		3.71 (-16.31–23.73)			
CD133^-^ /CD34^+^		21	11.64 ± 5.05	10.87 ± 3.24	7.11 ± 2.15	5.30 ± 2.46	0.092	0.854	0.737
	Change			-0.77 (-8.45–6.91)		-1.82 (-8.52–4.88)			
CD133^-^/ CD34^-^		21	32.93 ± 8.45	30.47 ± 5.49	26.13 ± 6.91	29.12 ± 7.45	0.610	0.938	0.708
	Change			-2.46 (-14.03–9.10)		2.99 (-3.71–9.68)			
CPC (CD34^+^)		29	0.08 ± 0.05	0.05 ± 0.02	0.06 ± 0.02	0.05 ± 0.02	0.630	0.419	0.633
	Change			-0.03 (-0.13–0.07)		-0.01 (-0.02–0.01)			

Parameters were evaluated before and after the aerobic exercise session. Data are expressed as mean ± standard deviation. Comparisons were evaluated using generalized estimating equations (GEE) with Bonferroni correction (n = between 10 and 29). Change means difference from post-exercise to pre-exercise (95% confidence interval).

* P < 0.001 *versus* pre-exercise values.

Abbreviations: CPC, circulating progenitor cells (expressed as a percentage of white blood cells [WBCs]); EPC, endothelial progenitor cells (expressed as a percentage of WBCs); TEC, total endothelial cells (expressed as a percentage of WBCs).

**Figure 3:**
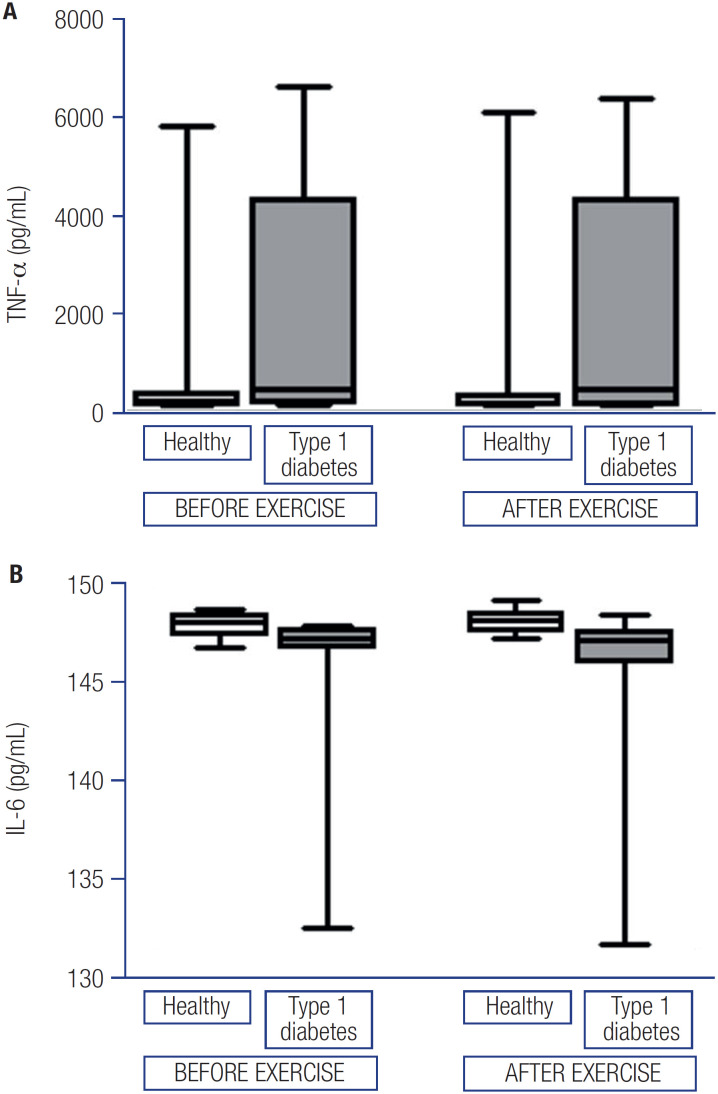
Levels of tumor necrosis factor-alpha (TNF-α) at baseline and after exercise (panel a). Levels of interleukin 6 (IL-6) at baseline and after exercise (panel b). Data are expressed as median and interquartile range. Comparisons were evaluated by generalized estimating equations (GEE) with Bonferroni correction. Individuals with type 1 diabetes (n = 15) and healthy individuals (n = 15). *P < 0.001 for healthy individuals versus those with type 1 diabetes.

Figure 3 shows the results of plasma TNF-α (panel A) and IL-6 (panel B) measurements. Levels of TNF-α before and after exercise were comparable in healthy individuals (210.2 [IQR 142.1-401.2] pg/mL and 191.3 [IQR 136.4-350.5] pg/mL, respectively) and in individuals with T1DM (463.8 [IQR 201.4-4306.0] pg/mL and 482.7 [IQR 143.8-4304.3] pg/mL, respectively; p group = 0.204, p time = 0.833, p interaction = 0.331). Similarly, levels of IL-6 before and after exercise were comparable in healthy individuals (148.2 [IQR 147.5-148.6] pg/mL and 148.2 [IQR 147.7-148.6] pg/mL, respectively) and in individuals with T1DM (147.2 [IQR 145.9-147.7] pg/mL and 147.2 [IQR 146.8-147.8] pg/mL, respectively; p group = 0.113, p time = 0.668, p interaction = 0.108).

## DISCUSSION

**Figure 4:**
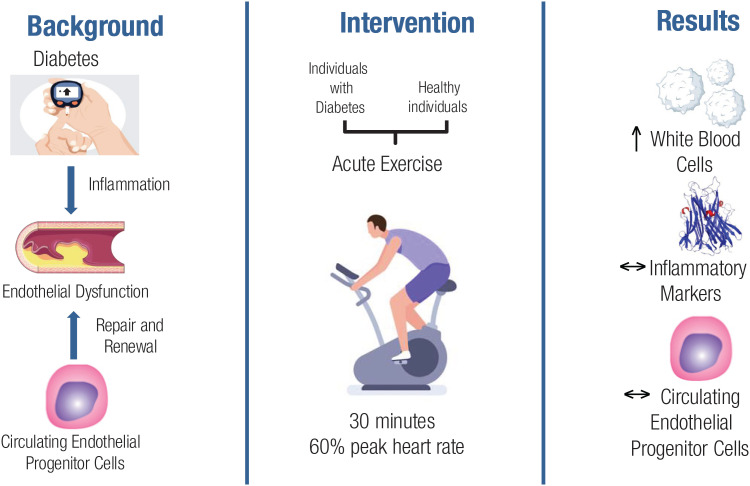
Graphical abstract depicting the overall implications of the study, along with the research framework.

The present study evaluated the effects of a single bout of submaximal aerobic exercise in patients with T1DM and healthy individuals and found no differences in circulating EPCs, TECs, or CPCs counts within and between groups before and after the exercise session. As expected, there was an increase in WBCs and neutrophils in both groups after the exercise. Notably, inflammatory markers remained unchanged after exercise in both groups. The overall implications of this study, along with the research framework, are summarized in the graphical abstract depicted in Figure 4, offering a concise visual representation of the results.

Comparing these findings with those from a previous study by our group, we found a similar result regarding baseline EPCs, *i.e.*, comparable values both in healthy individuals and in patients with T1DM. In contrast, our previous study found that EPCs reduced after exercise in healthy individuals, while the present study found no difference in EPC counts between the groups at this time point (17). In our previous study, EPCs were evaluated from peripheral blood mononuclear cells isolated using density gradient, whereas in the present study, they were analyzed directly in whole blood. Despite their clinical significance, circulating EPCs – identified by the CD34+/KDR+ immunophenotype – represent only ≤ 0.01% of all peripheral blood mononuclear cells; therefore, their assessment is technically challenging and requires high-sensitivity techniques (*e.g.*, flow cytometry) for their identification and quantification (21,24).

In the present study, where EPCs were identified by the CD34^+^/KDR^+^/CD45dim/^−^ immunophenotype, we found that EPC counts did not differ between patients with T1DM and healthy individuals and were not influenced by exercise. Interestingly, a previous study comparing control participants *versus* individuals with T1DM found a lower EPC count and a negative correlation (*r* = −0.429, p = 0.006) between glucose variability and EPC count when EPCs were identified by the CD34^+^/KDR^+^/CD133^+^ phenotype, but this finding was not confirmed when EPCs were identified by the CD34^+^/KDR^+^ immunophenotype (25). A study including adults with cardiovascular risk factors, which identified EPCs by the CD45^low^/CD34^+^/KDR^+^/CD133^+^/CD144^−^ immunophenotype found that a shorter (30-minute) bout of a multicomponent exercise, compared with a longer (45-minute) exercise session, promoted an acute increase in circulating levels of EPCs but no increase in levels of CPCs (26). The variability in results across studies, particularly regarding EPC responses to exercise, could be influenced by the different immunophenotypes used to identify EPCs. Differences in staining strategies may lead to variations in EPC detection, highlighting the need for standardized approaches when interpreting flow cytometric data. A recent systematic review evaluated 24 trials investigating the acute effects of different exercise durations on the numbers of circulating EPCs in healthy, nonclinical populations and found that only prolonged exercise (longer than 3 hours) elicited significant increases in EPC numbers in healthy individuals (16). Additionally, in patients with metabolic diseases (*e.g.*, diabetes) compared with healthy age-matched controls, an acute exercise bout has been shown to blunt EPCs recruitment (15).

The acute effects of the aerobic exercise session in increasing WBC and neutrophil counts in the present study is a well-described effect. This occurs due to the mobilization of leukocytes from marginal pools located in intravascular endothelial walls and from extravascular storage pools into the bloodstream in response to physiological stress, with exercise being one of the most potent stimuli (27,28). Considering that hematocrit levels remained stable 60 minutes after the exercise bout, and exercise-induced changes in plasma volume were not expected during this period (29), no correction for EPCs, WBCs, or neutrophils was necessary.

Surprisingly, there were no differences in the levels of the inflammatory markers TNF-α and IL-6 between healthy individuals and patients with T1DM at baseline or after exercise. Our results are aligned with those by Heier and cols., who found no difference in TNF-α and IL-6 levels between healthy controls and patients with T1DM, despite these markers correlating significantly with HbA1c levels in the T1DM group (30). West and cols. also observed that patients with T1DM with optimal glycemic control, despite having greater TNF-α concentrations, had comparable resting EPCs compared with healthy controls, but a blunted late post-exercise EPC response (31). In the present study, the differences could not be attributed to the glycemic control in the T1DM group, which did not have increased inflammatory level and had a mean HbA1c level of approximately 7.9%. Therefore, our findings suggest that the regulation of EPCs release in response to an exercise stimulus may be comparable between healthy individuals and patients with T1DM because of the lack of chronic inflammation.

We should acknowledge some limitations of our study. First, the intensity, duration, and type of exercise may not have been sufficient to trigger the level of endothelial damage required to elicit CPC/EPC mobilization. The exercise intensity was monitored by the participants’ heart rate, which, in the T1DM group, could have been influenced by cardiac changes associated with the natural course of T1DM and/or pharmacological treatment due to central nervous system impairment. Furthermore, we did not perform sequential assessments throughout the exercise session and recovery, and a potential turnover or clearance of EPCs and inflammatory markers in the face of the hemodynamic adjustments to the exercise session may have been missed. Finally, we may have overestimated the magnitude of the effect or underestimated the dispersion at the time of sample size calculation due to the nature of our sample characteristics, stimuli settings, and CPC/EPC responsiveness within this context. Finally, the sample size constrains additional analyses; therefore, any extrapolation of our findings should be approached with caution.

In conclusion, individuals with T1DM presented expected changes in blood count after a single aerobic exercise session, which was not accompanied by EPC or inflammatory changes. The comparison of EPCs between patients with T1DM and healthy individuals following an aerobic exercise session presents new research perspectives. Since the EPCs count was not significantly different between patients with T1DM and healthy individuals, both in rest and in response to exercise, it can be argued that inflammation may be the main factor explaining this finding. Therefore, the relationship between inflammatory markers and EPCs counts in patients with T1DM and healthy individuals needs to be investigated further in a study including a larger sample size.
